# TGF-β1 Suppresses Proliferation and Induces Differentiation in Human iPSC Neural *in vitro* Models

**DOI:** 10.3389/fcell.2020.571332

**Published:** 2020-10-28

**Authors:** Julia Izsak, Dzeneta Vizlin-Hodzic, Margarita Iljin, Joakim Strandberg, Janusz Jadasz, Thomas Olsson Bontell, Stephan Theiss, Eric Hanse, Hans Ågren, Keiko Funa, Sebastian Illes

**Affiliations:** ^1^Institute of Neuroscience and Physiology, Sahlgrenska Academy at University of Gothenburg, Gothenburg, Sweden; ^2^Oncology Laboratory, Department of Pathology, Sahlgrenska University Hospital, Gothenburg, Sweden; ^3^Department of Neurology, Heinrich-Heine-University, Düsseldorf, Germany; ^4^Department of Clinical Pathology and Cytology, Sahlgrenska University Hospital, Gothenburg, Sweden; ^5^Result Medical GmbH, Düsseldorf, Germany; ^6^Medical Faculty, Institute of Clinical Neuroscience and Medical Psychology, Heinrich Heine University, Düsseldorf, Germany; ^7^Section of Psychiatry and Neurochemistry, Institute of Neuroscience and Physiology, Sahlgrenska Academy at University of Gothenburg, Gothenburg, Sweden; ^8^Sahlgrenska Cancer Center, Institute of Biomedicine, Sahlgrenska Academy at University of Gothenburg, Gothenburg, Sweden

**Keywords:** human induced pluripotent stem cells, neural stem cells, neural differentiation, cortical development, TGF-β1

## Abstract

Persistent neural stem cell (NSC) proliferation is, among others, a hallmark of immaturity in human induced pluripotent stem cell (hiPSC)-based neural models. TGF-β1 is known to regulate NSCs *in vivo* during embryonic development in rodents. Here we examined the role of TGF-β1 as a potential candidate to promote *in vitro* differentiation of hiPSCs-derived NSCs and maturation of neuronal progenies. We present that TGF-β1 is specifically present in early phases of human fetal brain development. We applied confocal imaging and electrophysiological assessment in hiPSC-NSC and 3D neural *in vitro* models and demonstrate that TGF-β1 is a signaling protein, which specifically suppresses proliferation, enhances neuronal and glial differentiation, without effecting neuronal maturation. Moreover, we demonstrate that TGF-β1 is equally efficient in enhancing neuronal differentiation of human NSCs as an artificial synthetic small molecule. The presented approach provides a proof-of-concept to replace artificial small molecules with more physiological signaling factors, which paves the way to improve the physiological relevance of human neural developmental *in vitro* models.

## Introduction

Human induced pluripotent stem cell (hiPSC)-based two-dimensional (2D) and more complex three-dimensional (3D) neural *in vitro* models offer the opportunity to study the principles of human brain development and neuronal circuit function (Shi et al., 2012; Lancaster et al., 2013; Paşca et al., 2015). The major consecutive neural developmental phases are: induction of neural identity in human pluripotent stem cells to differentiate into neural stem cells (NSCs); proliferation and regional specification; neuronal and glial commitment; cell-cycle exit of NSCs, followed by either cell death, differentiation into neurons or into glial cells. The further maturation of post-mitotic neurons and glial cells are leading to the formation of functional neuronal circuits. However, hiPSC neural *in vitro* models represent fetal rather than adult brain tissue properties, such as on-going proliferation and a lengthy process of neuronal and glial maturation. Since plethora of animal-based studies demonstrated that neurotrophic factors, e.g., brain derived neurotrophic factor (BDNF), glia derived neurotrophic factor (GDNF), and neurotrophin-3 (NT-3), are crucial for neuronal survival, neurite growth and synapse development, these physiologically relevant factors are commonly used to support late stages of neural development in human iPSC neural models. In contrast, small molecules, such as DAPT and PD0332991, are not physiologically relevant factors, but commonly applied to promote early processes of neural development in human iPSC neural models, e.g., the transition of NSCs into neurons (Kirkeby et al., 2012; Kemp et al., 2016). Thus, we aimed to identify and evaluate physiologically relevant factors that promote the transition of NSCs into neurons in human iPSC-derived neural *in vitro* models.

Recently, we demonstrated that human cerebrospinal fluid (CSF) obtained from healthy adult individuals caused several maturation processes, including the rapid transition of NSCs into neurons and astrocytes in a human iPSC 3D neural aggregate *in vitro* model (Izsak et al., 2020). This demonstrates that a human iPSC 3D neural aggregate *in vitro* model can adopt rather mature properties when exposed to an appropriate and physiologically relevant environment. The use of fetal human CSF would allow the creation of a more physiological *in vitro* differentiation environment for hiPSC NSC models. CSF sampling in intrauterine human fetal brain would enable the identification of specific physiologically relevant neural differentiation factors. Since this approach is virtually impossible to achieve due to ethical concerns, other approaches are needed.

To circumvent these limitations, we performed literature study and used data from the human transcriptome database (Kang et al., 2011) to identify known signaling proteins that are specifically up-regulated during the early phases of cortical development and become down-regulated reciprocal to the upregulation of genes involved in neuronal and glial development. We surmised that those signaling proteins might mediate the transition of neural stem cells into neurons or astrocytes in human iPSC-derived neural *in vitro* models.

## Results

### TGF-β Is a Potential Physiological Regulator of Neural Stem Cell Proliferation, Neuronal, and Glial Differentiation During Early Human Fetal Cortical Development

**FIGURE 1:**
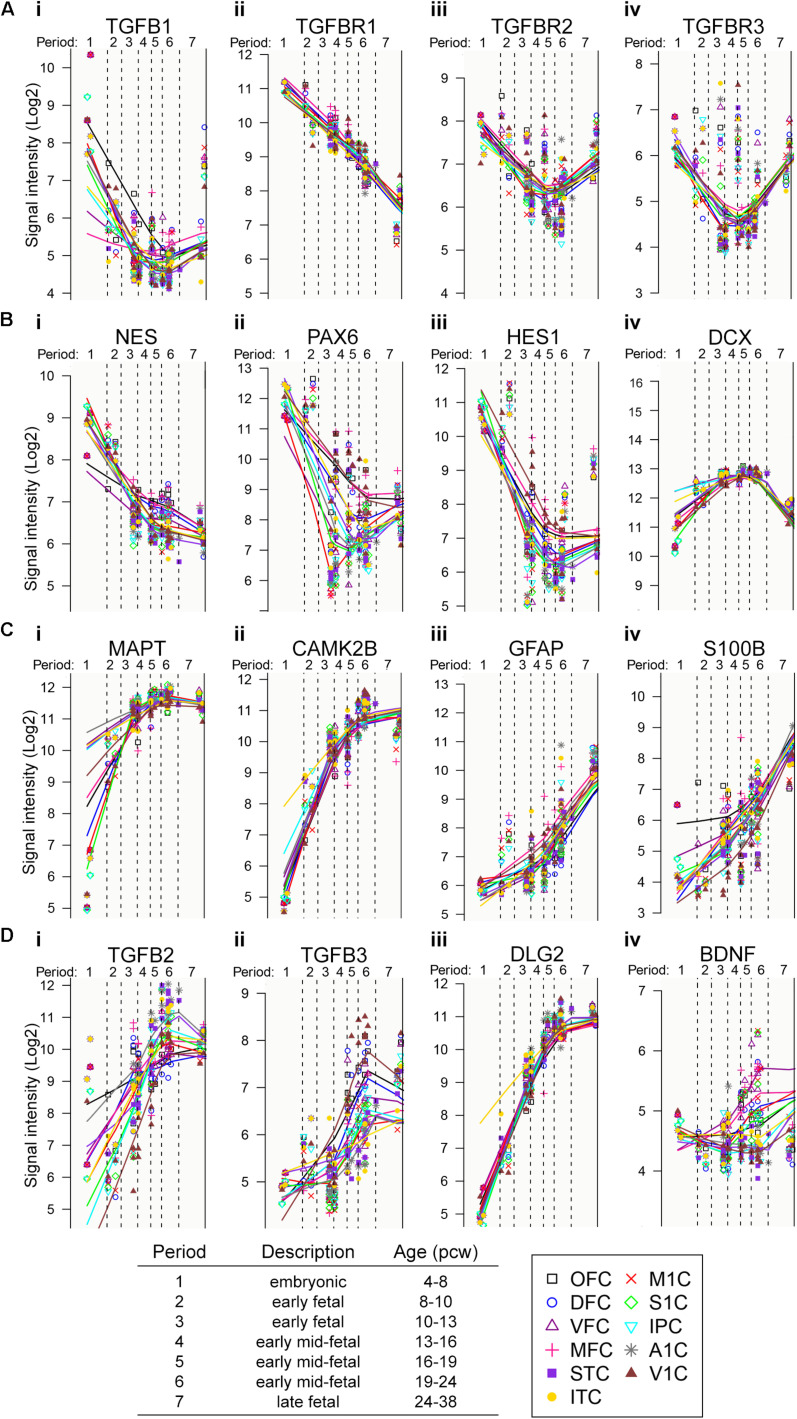
Evidence for relevance of TGF-β signaling in human fetal development. **(A–D)** Gene-expression profile of transcripts of interest during human prenatal cortical development.

Based on accumulated evidence in rodent models on the role of TGF-β on neuronal development (Vogel et al., 1991; Aigner and Bogdahn, 2008; Watabe and Miyazono, 2009; Kandasamy et al., 2011; Kraus et al., 2013; Caraci et al., 2015), we assessed the patterns of expression of TGF-β signaling components (ligands and receptors) in human embryonic and fetal brain development, using the human transcriptome database available at http://hbatlas.org/ (Kang et al., 2011; Figure 1). Gene expression profiling of different parts of human fetal cortex tissues revealed higher expression of TGF-β1 and TGF-β RI, RII, and RIII during the fourth post-conception week (pcw) compared to pcw 16–19, showing a progressive decrease of gene expression (Figures 1Ai–iv). This expression pattern is similar to specific NSC transcripts, such as nestin (NES), pax-6 and hes-1 (Figures 1Bi–iii) and it is reciprocal to the gene expression of doublecortin (DCX) (Figure 1Biv), a gene expressed by neuronal progenitor cells, and neuron-related genes, such as axonal protein Tau (MAPT) (Fiock et al., 2020) and CamKinase (CamK2b) (Wayman et al., 2008; Figures 1Ci,ii), as well as astrocytes-related genes, such as GFAP and S100beta (Figures 1Ciii,iv). In contrast, TGF-β2 and β3 are expressed at lower levels during the earlier stages of human fetal cortex development (before pcw 10–13) compared to pcw 19–24, with a gradual up-regulation starting after pcw 10–13 reaching their maximum expression at late fetal stages (24 till 38 pcw) (Figures 1Di,ii) which is comparable to the genes involved in synaptogenesis, such as post-synaptic density protein [PSD-93 aka DLG2 (disc large homolog2)] and BDNF (Figures 1Diii,iv). These data suggest that TGF-β1 has its exert function during very early fetal developmental stages (pcw 4–8), and thereby, might play an instructive role in NSC development during earlier fetal stages of human corticogenesis (pcw 4 till 8), a role that, however, has not been directly assessed.

### TGF-β Ligands and Receptors Are Present in hiPSC-Derived Neural Stem Cells

**FIGURE 2:**
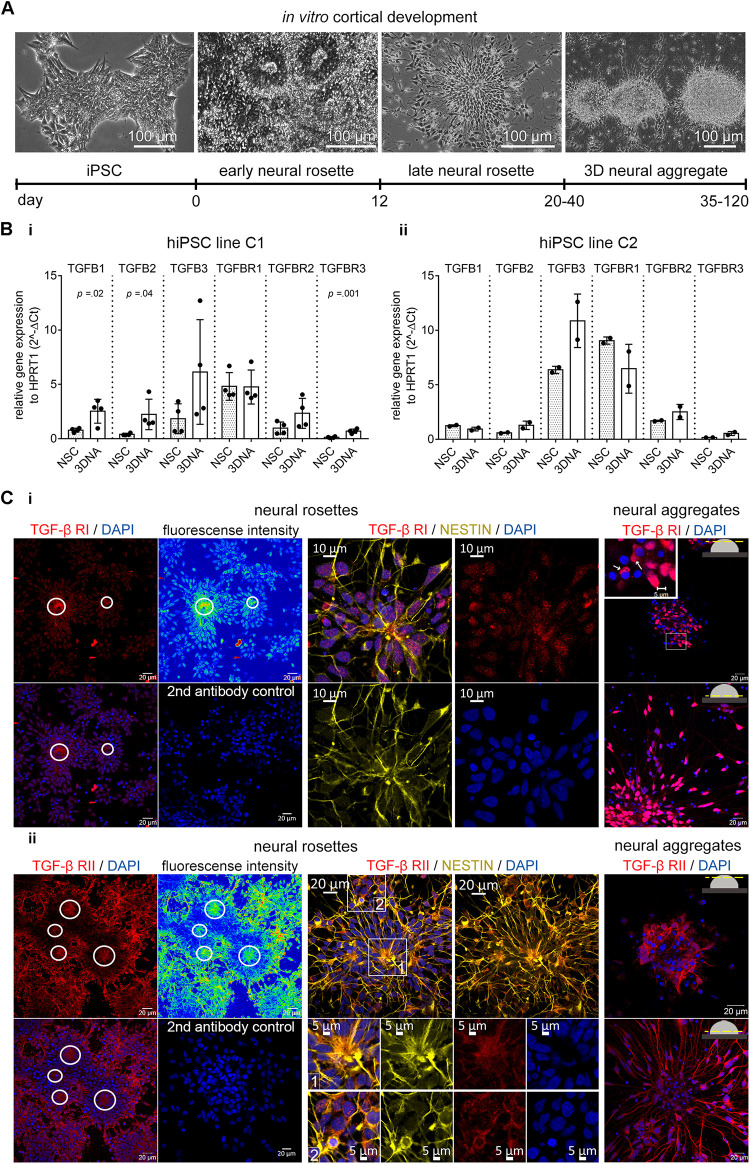
TGF-β receptors and ligands in human iPSC-derived neural *in vitro* model. **(A)** Phase contrast images and related timeline show the *in vitro* cortical development from hiPSCs toward 3D neural aggregates. **(B)** Gene expression of TGFB ligands and receptors in human iPSC-derived neural rosette (NSC) and neural aggregate (3DNA) cultures in **(i)** hiPSC line C1 and **(ii)** hiPSC line C2. The data is presented as mean ± standard deviation, unpaired *t*-test was used to calculate the indicated *p*-values. *N* = 1–2/cell line, each dot represents the value from one technical replicate. **(C)** Confocal images of **(i)** TGF-β1 RI and **(ii)** RII in the NESTIN^+^ cells in neural rosettes (left) and in the neural aggregates (right). Color coded fluorescent density images and circles highlight the accumulation of TGF-β receptors at the luminal side of neural rosettes. The secondary antibody control images are also shown (2nd antibody control). Insets highlight the cytoplasmic localization of TGF-β RI and II.

By using the commonly applied “dual-SMAD-inhibition” protocol for neural differentiation of hiPSC (Shi et al., 2012; Figure 2A), we generated hiPSC-derived NSC cultures with dorsal telencephalic identity (Supplementary Figure 1). Nestin^+^/PAX-6^+^ NSCs grow either as neural rosettes, where NSCs show strong CD133 expression at the luminal side of neural rosettes or NSCs are organized as adherently growing 3D neural aggregates (3D-NAs) (Supplementary Figure 1, the 3D-NAs are marked with boxes). As reported previously (Edri et al., 2015; Izsak et al., 2019), neural rosettes progressively give rise to 3D-NAs (Figure 2A).

We evaluated the expression of TGF-β ligands and receptors in human iPSC-NSC and 3D-NA cultures and confirmed their presence by qPCR (Figure 2B). We further evaluated the appearance of the TGF-β receptors by immunocytochemistry and confocal microscopy. The TGF-β RI and RII proteins were present in Nestin^+^- NSCs at neural rosette stage (Figures 2Ci,ii, neural rosettes, marked by circles) as well as at 3D neural aggregate stage (Figures 2Ci,ii, neural aggregates).

### TGF-β1 Suppresses Proliferation, Enhances Neuronal, and Glial Differentiation in Human iPSC Neural Stem Cell Cultures

We surmised that endogenously produced TGF-β influences proliferation and differentiation processes. Furthermore, we surmised that the endogenously produced amount of TGF-β might not be enough to completely suppress proliferation and enhance differentiation. TGF-β1 is produced by the embryonic choroid plexus and secreted into embryonic CSF (Falk et al., 2008). Thus, we studied whether additionally applied TGF-β1 influences proliferation as well as neuronal and glial differentiation in hiPSC-derived NSC and 3D-NA cultures.

**FIGURE 3:**
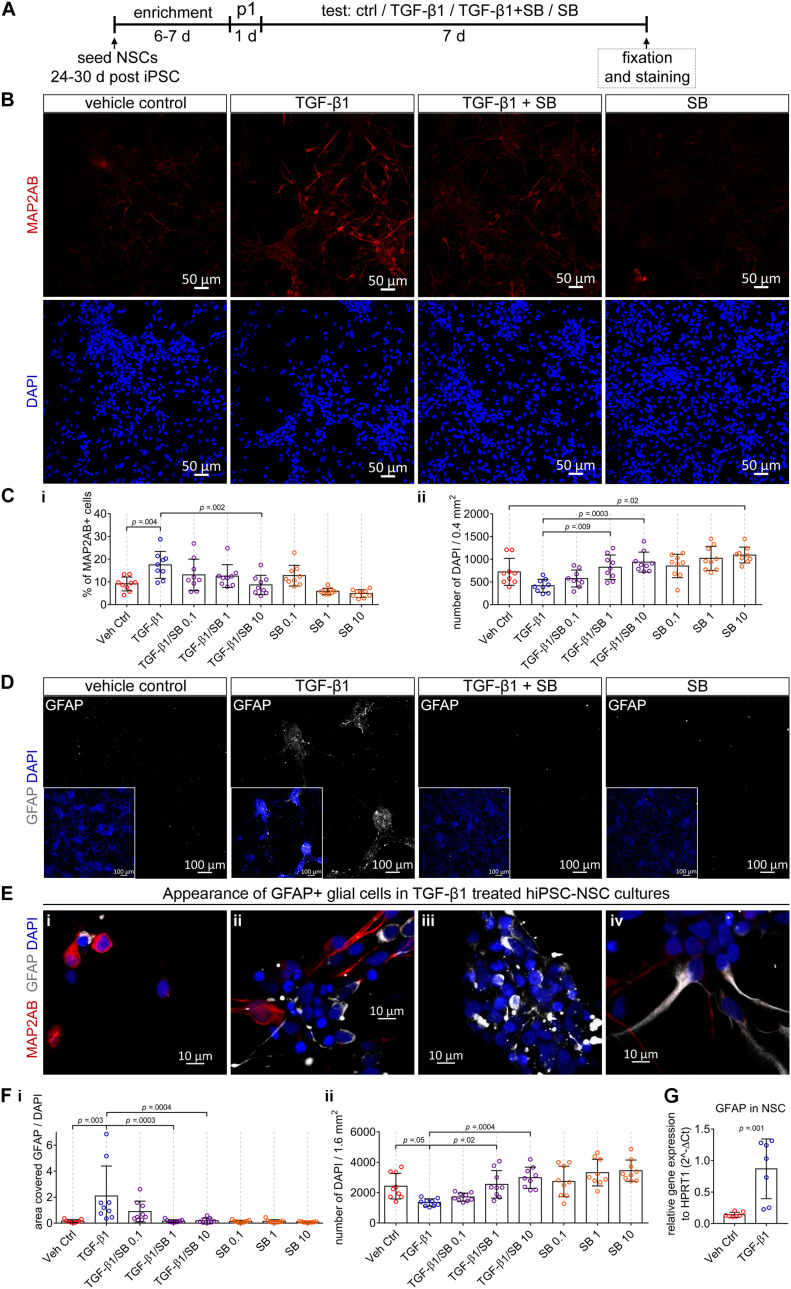
The impact of TGF-β1 in human iPSC-derived neural stem cell cultures. **(A)** Schematic representation of the experimental design. **(B)** Confocal images visualize MAP2AB^+^ neurons in vehicle control, TGF-β1, TGF-β1 + SB, and SB treated hiPSC-NSC cultures. **(C)** Diagrams show the percentage of MAP2AB^+^ neurons **(i)** and the number of DAPI cells **(ii)** quantified in the cultures in the indicated conditions (hiPSC line C1, *N* = 3, *n* = 2–3). **(D)** Example overview images (10x) visualizing GFAP^+^ glial cells in vehicle control, TGF-β1, TGF-β1 + SB, and SB treated hiPSC-NSC cultures. **(E)** Example confocal images (40x) visualize the appearance of GFAP^+^ glial cells among MAP2AB^+^ neurons, in TGF-β1 treated hiPSC-NSC cultures. **(F)** Diagrams show **(i)** the area covered by GFAP^+^ glial cells and **(ii)** the number of DAPI cells quantified in the cultures under the indicated conditions (hiPSC line C1, *N* = 3, *n* = 3). **(G)** Gene expression of GFAP in vehicle control and TGF-β1 treated cultures (hiPSC line C1 and C2, *N* = 2, *n* = 1–2). The data is presented as mean ± standard deviation. Either one-way ANOVA with Tukey’s correction (multiple comparisons) or unpaired *t*-test was used to calculate the indicated *p-*values.

For this purpose, we exposed NSC cultures for 7 days to (i) TGF-β1, (ii) TGF-β1 together with the small-molecule SB 431542 (blocker of TGF-β signaling) or (iii) SB 431542 alone (Figure 3).

We revealed that TGF-β1 treatment results in an increase in the number of MAP2AB^+^ neurons (Figures 3B,Ci) and decrease of total number of cells (Figure 3Cii). Application of SB 431542 to TGF-β1-treated cultures, led to a concentration-dependent reduction of TGF-β1-mediated increase in the number of MAP2AB^+^ neurons (Figure 3Ci) and decrease of total number of cells (Figure 3Cii). Complementary, solely application of SB 431542 to human iPSC-derived NSC cultures induced a concentration-dependent increase in the total number of cells and led to a reduction in the number of MAP2AB^+^ neurons (Figure 3C).

Next, we assessed whether TGF-β1 influences glial development. We observed that GFAP^+^-glial cells were nearly absent in untreated conditions (Figure 3D, vehicle control). However, application of TGF-β1 led to more GFAP^+^-glial cells (Figure 3D, TGF-β1), where most GFAP^+^-glial cells showed a perinuclear localization (Figures 3Ei–iii) and we observed only occasionally cells with GFAP in cellular processes (Figure 3Eiv). Since GFAP^+^-glial cells were nearly absent in vehicle control-treated, TGF-β1/SB 431542 co-treated and SB 431542-treated cultures (Figure 3D), we did not count the number of GFAP cells but rather assessed the combined total area [mm^2^] covered by GFAP-signal normalized to the total number of DAPI-stained cells. By this, we confirmed that only TGF-β1-treated cultures showed a good detectable amount of GFAP-signal and blocking of TGF-β signaling using SB 431542 led to a concentration-dependent reduction of GFAP-signal (Figure 3Fi). Since the GFAP^+^-glial cells were nearly absent in untreated conditions, we could not detect differences in GFAP^+^-glial cells in SB 431542-treated cultures (Figure 3Fi). Complementary performed QPCR analysis confirmed that TGF-β1 application led to strong increase of GFAP expression (Figure 3G).

Comparable results were obtained by using an additional hiPSC line (Supplementary Figure 2).

These data demonstrate that (i) endogenously present TGF-β signaling regulates proliferation and neuronal differentiation, (ii) additionally applied TGF-β1 suppresses proliferation and enhances neuronal and glial differentiation in human iPSC-derived NSC cultures and that (iii) TGF-β signaling regulates proliferation, neuronal and glial differentiation in human iPSC-NSC cultures.

### TGF-β1 Suppresses Proliferation and Enhances Neuronal Differentiation in Human iPSC 3D Neural Aggregate Cultures

**FIGURE 4:**
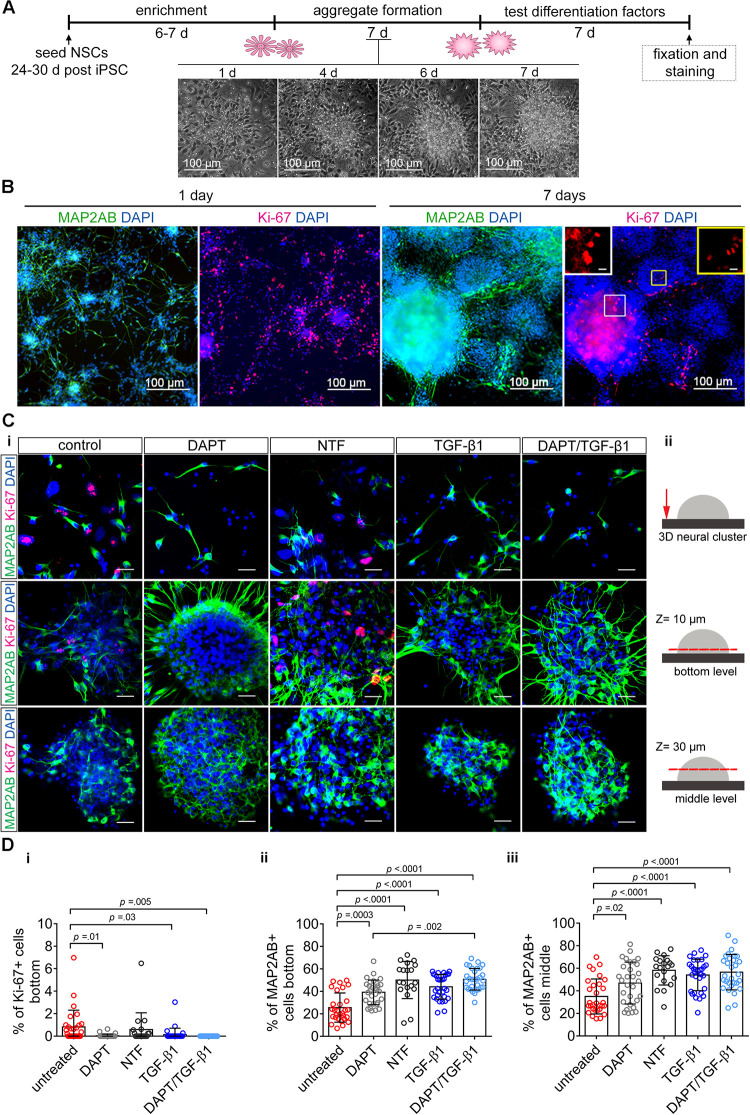
The impact of TGF-β1 on human iPSC-derived 3D neural aggregates. **(A)** Schematic representation of the experimental design. Time-lapse imaging shows the transition of neural rosette toward 3D-neural aggregate within 7 days. **(B)** Confocal images visualize MAP2-AB^+^-neurons and Ki-67^+^-cells in neural rosettes and 3D-neural aggregates (day 30 post iPSC stage). Insets show Ki-67^+^-cells at higher magnification. **(Ci)** Detailed confocal images visualize MAP2AB^+^-neurons and Ki-67^+^-proliferating cells in untreated, DAPT, neurotrophic factor mix (see main text), and TGF-β1 and DAPT/TGF-β1 treated hiPSC-NSC cultures. Scale bar: 20 μm. **(Cii)** Schematic drawings illustrate the regions and z-levels of image acquisition. **(D)** Diagrams show the percentage of **(i)** Ki-67^+^-cells and **(ii,iii)** MAP2AB^+^-neurons under the indicated culture conditions. Data are shown as mean ± standard deviation, one-way ANOVA with Tukey’s correction (multiple comparisons) was used to calculate the indicated *p*-values (line ChiPS4, *N* = 3, each dot represents one aggregate, taken from 2 to 3 technical replicates).

Neural stem cells cultured in adherent conditions *in vitro* form neural rosettes and progressively give rise to neural aggregates (Figures 4A,B). With time, NSCs undergo spontaneous neuronal differentiation indicated by the presence of MAP-2AB^+^ neurons in NSC and 3D-NA cultures (Figure 4B). However, morphological analyses indicate on-going proliferation, which was further confirmed by the presence of Ki67^+^ cells (Figure 4B). These residing NSCs and proliferation lead to overgrowth and detachment from the surface in adherent culture conditions. Thus, we analyzed whether the application of TGF-β1 suppresses proliferation and enhances differentiation also in a human iPSC 3D neural culture model.

Since we observed that proliferating NSC and differentiated neurons are located in different 3D levels in 3D-NAs cultures, we decided to perform a quantification analysis at the bottom and in the middle of 3D-NAs. In detail, we quantified the number of Ki-67^+^-proliferative cells and MAP2AB^+^ neurons in untreated and TGF-β1-treated human iPSC-derived neural cultures 7 days after treatment (Figures 4C,D). In addition, we used two positive control conditions to evaluate the efficiency of TGF-β1 in regulating proliferation and neuronal differentiation. DAPT is commonly applied inhibitor of NOTCH-signaling and reported to suppress proliferation and enhance neuronal differentiation in human NSCs *in vitro* (Borghese et al., 2010). Since neurotrophic factors BDNF, GDNF, NT-3, and FGF18 (in the following referred to as NTF) are commonly used to enhance neuronal differentiation *in vitro*, we used a neurotrophic factor mix as another positive control (Figures 4C,D).

Quantification of Ki67^+^-proliferating cells revealed that only TGF-β1-treated and DAPT-treated cultures show a significant reduction in the number of Ki67^+^-proliferating cells in comparison to untreated control (Figure 4Di). However, neurotrophic factors were insufficient to suppress proliferation. Co-application of DAPT/TGF-β1 abolished all proliferation, as indicated by the absence of Ki67^+^-proliferating cells (Figure 4Di).

Quantification of MAP2AB^+^-neurons revealed that all tested differentiation conditions enhanced neuronal differentiation in comparison to untreated hiPSC-NSC cultures (Figures 4Dii,iii). TGF-β1- and DAPT-treated cultures showed a similar increase in the percentage of MAP2AB^+^-neurons. Co-application of DAPT/TGF-β1 and also the mixture of different neurotrophic factors significantly increased the number of neurons (Figures 4Dii,iii).

Note, no significant difference could be found between DAPT and TGF-β1 treated cultures in respect of reducing proliferation and increasing neuronal differentiation.

This finding shows that additionally applied TGF-β1 is suitable to suppress proliferation and to enhance neuronal differentiation in human iPSC 3D neural aggregate cultures. Moreover, the presented data represent a proof-of-concept that artificial small molecules, such as gamma secretase inhibitors, can be replaced by physiological signaling factors, e.g., TGF-β1.

### TGF-β1 Does Not Alter Electrophysiological Function of Human iPSC-Derived Neurons

**FIGURE 5:**
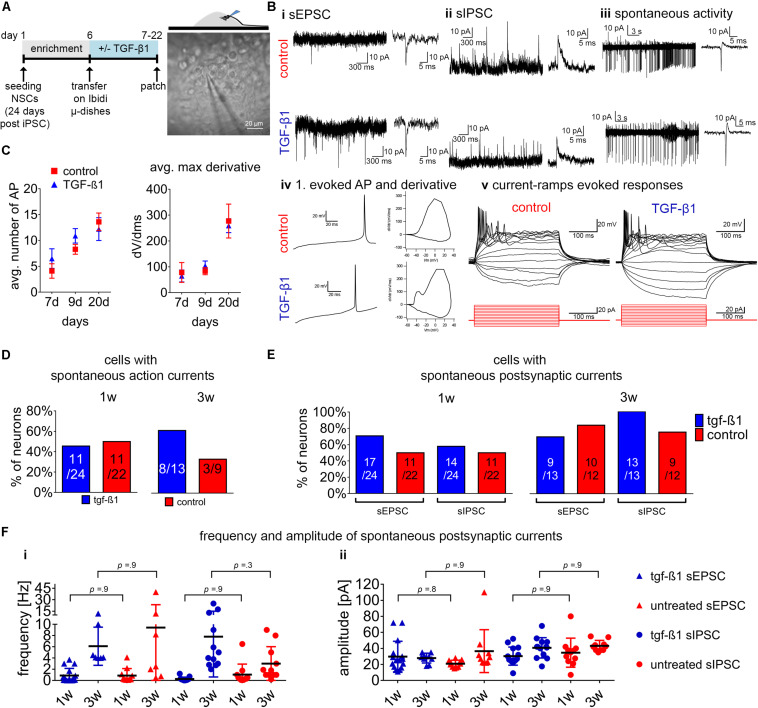
Assessment of TGF-β1 in functional neuronal maturation. **(A)** Schematic representation of the experimental design on the left and phase-contrast image on the right illustrating the position of the patch-clamp electrode at 3D-neural aggregates to assesses the electrophysiological functionality in untreated and TGF-β1 treated hiPSC-NSC cultures. **(B)** Examples of recorded spontaneous synaptic **(i)** excitatory post-synaptic currents (EPSC), **(ii)** inhibitory post-synaptic currents (IPSCs), **(iii)** spontaneous action currents and **(iv)** first evoked action potential and phase-plan plots, and **(v)** example sweeps showing evoked action potentials in response to depolarizing current pulses in cells. Visualized injected current pulses (300 ms) starting from –20 toward 50 pA (15 pulses with steps of 5 pA, from hyperpolarizing to depolarizing) from the holding current. **(C)** Diagrams show the average number of action potentials and average max derivatives recorded at 1 and 3 weeks of cultivation. **(D)** Diagram presents the percentage of neurons showing spontaneous action currents after 1 and 3 weeks in culture. **(E)** Diagrams show the percentage of neurons showing spontaneous postsynaptic currents, and **(F)** frequency and amplitude of spontaneous postsynaptic currents recorded after 1 and 3 weeks in culture. Data are shown as mean ± standard deviation, one-way ANOVA with Tukey’s correction (multiple comparisons) was used to calculate the indicated *p*-values (line ChiPS4, *N* = 2, *n* = 2–3).

As presented previously using murine (Illes et al., 2009) and human pluripotent stem cell-derived 3D-NA models (Izsak et al., 2019), neurons are electrophysiologically active and form functional neuronal networks in 3D neural models. The TGF-β1 impact on human neural cells has not yet been reported, therefore we evaluated whether TGF-β1 treatment affects electrophysiological function of human neurons. Since human iPSC-derived neurons are more functional in an 3D neural environment (see Izsak et al., 2019, 2020), we prepared hiPSC-3D-NA cultures in the absence or presence of 20 ng/ml TGF-β1. We performed cell-attached and whole cell recordings in cells localized at the edges of 3D-NAs, where MAP2AB^+^ neurons are present (Figure 5A). Infrared differential interference contrast video microscopy was used to identify the localization of cells with neuronal appearance (Figure 5A). We assessed the electrophysiological properties of cells after the first week (days 7–9) and third week (days 20–22) of cultivation. In total 71 cells were successfully patched and analyzed.

Neurons with excitatory and inhibitory post-synaptic currents (Figures 5Bi,ii) could be found in 3D-NAs from both TGF-β1-treated and non-treated cultures. These neurons generated spontaneous action potentials and bursting activity (Figure 5Biii), and exhibited evoked spiking and bursting (Figures 5Biv,v).

We used the maximal number of evoked action potentials (Figure 5C) and the maximal rate of change (d*V*/d*t*) as parameter to describe neuronal maturation over time. From the first to the third week of cultivation, neurons in TGF-β1-treated and non-treated cultures showed a nearly identical increase of maximal number of evoked action potentials (Figure 5C) and of the maximal rate of change (d*V*/d*t*) of the first evoked action potential (Figure 5C). After 3 weeks of cultivation, neurons within 3D-NAs showed evoked high-frequency bursting and high values of the maximal rate of change (d*V*/d*t*) of the first evoked action potential that is typical for functional neurons (Bardy et al., 2016).

Next, we compared the number of cells showing these electrophysiological properties in TGF-β1-treated and non-treated groups at different time points. After 3 weeks of cultivation, we found more neurons showing spontaneous action currents in the TGF-β1-treated than in the non-treated group (Figure 5D). During the first week of differentiation, the number of neurons showing spontaneous synaptic activity was slightly higher in TGF-β1-treated cultures (Figure 5E). However, at the third week of differentiation there was only a slightly increased number of neurons with spontaneous IPSC in the TGF-β1-treated cultures.

To describe synapse development over time, we analyzed the frequency and amplitude of spontaneous synaptic activity. We observed that the mean frequency of postsynaptic excitatory and inhibitory currents increased from the first to the third week of cultivation (Figure 5Fi), while the mean amplitude remained unchanged (Figure 5Fii). However, our analysis did not reveal any differences between TGF-β1 treated and untreated cultures.

**FIGURE 6:**
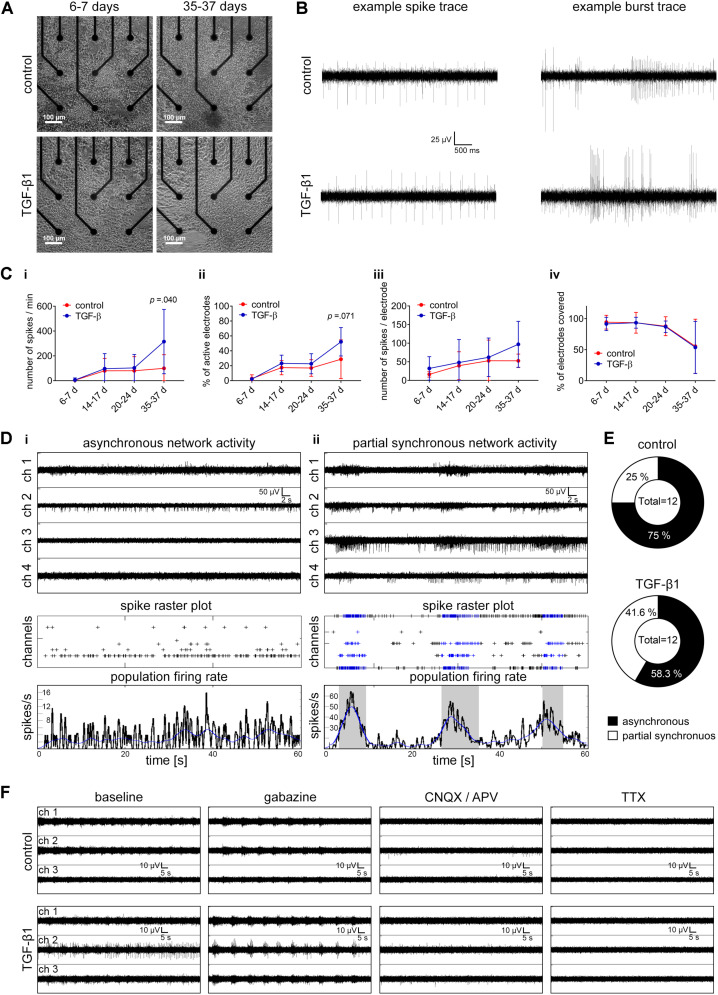
Assessment of TGF-β1 in functional neuronal circuit formation. (A) Phase-contrast images show the morphology of TGF-β1 treated and untreated hiPSC-neural cells cultured on a nine-electrode array of a six-well MEA for 1 week and 5 weeks. **(B)** Example traces of spiking and bursting activity recorded in TGF-β1 treated and untreated hiPSC-neural cultures. **(C)** Diagrams illustrate neuronal population activity parameters in TGF-β1-treated and untreated hiPSC-neural cultures. Data are presented as mean ± SD. Unpaired *t*-test between the groups was applied to calculate the indicated *p*-values (hiPSC line C2 and ChiPS4, *N* = 2–3, *n* = 6 per condition per line). **(D)** Representative examples of MEA recordings, spike raster plots (SRP) and population-firing rates illustrate the activity of asynchronously active **(i)**, and partially synchronously active **(ii)** neuronal populations. **(E)** Pie charts illustrate the percentage of hiPSC-neural cultures showing asynchronous or partially synchronous neuronal activity in TGF-β1 treated and untreated groups. **(F)** Representative examples of MEA recordings showing neuronal activity of TGF-β1 treated and untreated hiPSC-neural cultures before and after Gabazine, CNQX, AP-5, and TTX-treatment, respectively.

In addition, we cultured hiPSC-NSCs on microelectrode arrays and compared the neuronal network development of TGF-β1 treated and untreated cultures up to 5 weeks (Figure 6).

Under both conditions, neural cultures showed spiking and bursting activity within 2 weeks in culture (Figure 6B). The number of spikes and the number of electrodes detecting spike activity increased within the following 3 weeks (Figure 6C). After 35–37 days *in vitro*, TGF-β1 treated neural cultures showed a significant higher number of spikes (Figure 6Ci), and, although not significant, a higher number of spike-detecting electrodes in comparison to untreated control cultures (Figure 6Cii). By normalizing the number of spikes to the number of spiking activity-detecting electrodes, TGF-β1-treated neural cultures showed higher, although not significant, normalized spike activity over a time period of 5 weeks (Figure 6Ciii). Weekly visual inspection of TGF-β1-treated and untreated neural cultures over this time period revealed no differences in number of electrodes covered by cells (Figure 6Civ).

Under both conditions, neuronal cells formed a functional neuronal network within 35–37 days *in vitro*, that were either asynchronously or partially synchronously active (Figure 6D). Interestingly, we observed more partially synchronously active neuronal populations in the TGF-β1 treated neural cultures (Figure 6E). Neither untreated nor TGF-β1 treated neural cultures showed highly synchronous neuronal population activity. However, the formation of a functional interconnected neuronal population was confirmed by induction of highly synchronous population burst activity mediated by inhibition of the GABA_A_-receptor through gabazine treatment (Figure 6F). The gabazine-induced highly synchronized activity was absent after the application of NMDA and AMPA receptor antagonists (Figure 6F, CNQX/APV), and the residual spiking activity was abolished after application of the voltage-gated sodium channel blocker Tetrodotoxin (TTX) (Figure 6F).

## Discussion

Human pluripotent stem cell-derived neural *in vitro* models are used to obtain insight into human neural development and neuronal circuit formation. A commonly used approach to study human neural development *in vitro* is based on 3D neural models, such as 3D neural spheroids or brain organoids, in which no additional factors are applied to the culture media to avoid external manipulation of *in vitro* human neural development processes. Moreover, the application of external factors to 3D neural spheroids or brain organoids is rather considered as artificial modulation of human neural development to enhance neuronal or glial development *in vitro*. However, the human fetal brain is not an isolated organ and peripheral signaling via blood-choroid plexus-cerebrospinal fluid signaling path occur and is essential to synchronize the development of the brain with the development of other organs.

### The Role of Choroid Plexus and CSF Factors in Regulating Neural Development

It has been suggested and shown that hCSF composition besides its function in supporting homeostasis, regulates neural development and function by secreted factors. During fetal brain development and aging, the composition of the CSF is changing and how CSF factors regulate stem cell and progenitor proliferation as well as neurogenesis is age-dependent (Gato and Desmond, 2009; Lehtinen et al., 2011; Johansson, 2014; Silva-Vargas et al., 2016; Ghersi-Egea et al., 2018). Animal based studies showed that TGF-β1 produced by the choroid plexus is secreted into the cerebrospinal fluid where it becomes distributed by the ventricular system and finally binds to TGF-β receptor expressing NSCs within embryonic mouse brain (Falk et al., 2008). During fetal brain development, CSF factors might be crucial to promote differentiation of NSCs into neurons, while in the adult brain, CSF factors rather promote proliferation of ventricular NSCs (Silva-Vargas et al., 2016). Interestingly, human iPSC-NSCs differentiate into neurons and astrocytes when exposed to adult CSF samples (Izsak et al., 2020).

In the here presented work, we provide a proof-of-concept that (i) literature study together with available human transcriptome databases containing gene-expression profiles from fetal and adult tissue samples allow the identification of prospective neural developmental factors, and (ii) that human iPSC-derived NSC and complex 3D neural aggregate cultures allow the validation of candidate signaling factors in their capacity of regulating human NSC development, neuronal function and neuronal circuit formation. While animal-based studies revealed that the here selected candidate signaling factor TGF-β1 regulates various processes during rodent brain development, it was unknown if TGF-β signaling is essential for human neural development *in vitro*. In respect to human iPSC-based neural models, TGF-β signaling was only known for its role in promoting neural induction, i.e., preventing endodermal development and promoting ectodermal development of pluripotent stem cells. However, if TGF-β1 influences human NSC development, neuronal function and neuronal circuit formation was unknown.

### TGF-β Signaling in Regulating Neural Stem Cell Proliferation

Here, we demonstrate that TGF-β signaling is endogenously present in human iPSC- NSC cultures and represent an autocrine negative regulator for proliferation. In 3D-NA cultures, TGF-β1 treatment results in a similar reduction of proliferating cells as observed after DAPT treatment. However, co-application of TGF-β1 and DAPT is required to obtain neural cultures where proliferation is absent. A possible explanation for this might be that the neural stem and progenitor cell population is heterogeneous and that proliferation of some neural stem and progenitor cells can be modulated by TGF-β1 signaling, while proliferation of other neural stem and progenitor cells depends on NOTCH-signaling.

Since animal-based *in vitro* and *in vivo* models demonstrate that TGF-β1 is a suppressor of NSC proliferation (e.g., Wachs et al., 2006; Stipursky et al., 2014), our data provides evidence that TGF-β1 is an important signaling cue also for the regulation of human NSC proliferation.

### TGF-β Signaling in Neuronal Differentiation of Human Neural Stem Cells

The transition of NSCs into neurons represent the first step of neuronal differentiation. We demonstrate that TGF-β signaling represents an autocrine positive regulator for neuronal differentiation in human iPSC-NSC cultures. Furthermore, we demonstrate that additionally applied TGF-β1 further promotes neuronal differentiation, which provide further evidences that the amount of endogenously produced TGF-β1 in a human neural stem population is not sufficient and neurogenesis required external or extracerebral sources of TGF-β1. Supporting data for this hypothesis were presented by Stipursky et al. (2014). Stipursky et al. (2014) demonstrating that intraventricular injection of TGF-β1 into embryonic brain results into a strong increase of number of βTubulin3-positive neurons in embryonic cortex [see Figure 4 in Stipursky et al. (2014)]. Using 3D-NA cultures, we demonstrate that TGF-β1 treatment results in a similar increase of neurons as observed after DAPT treatment. However, co-application of TGF-β1 and DAPT does not have an additive effect and does not further increase the number of neurons. This data demonstrates that artificial small compounds, e.g., DAPT, can be replaced by physiological relevant signaling cues, e.g. TGF-β1, to promote and study human neurogenesis *in vitro*.

### TGF-β Signaling in Glial Differentiation of Human Neural Stem Cells

As indicated by the transcript profile of neuronal and astrocyte-related transcripts during human fetal brain development (Figure 1), and in line with established knowledge, human astrocyte development follows human neurogenesis. Thus, it was surprising for us to observe that additional application of TGF-β1 induces the appearance of GFAP^+^-cells in early human NSC cultures (30–40 days post-iPSC stage). However, those TGF-β1-induced GFAP-cells did not have a stellate, elongated or flat morphology as regular *in vitro* astrocytes have. Nearly all TGF-β1-induced GFAP-cells, and the very few GFAP-cells in untreated human NSC cultures, have a perinuclear localization of GFAP reminiscent of an immature glial cell. Interestingly, intraventricular injection of TGF-β1 into E14 embryonic mouse brain (note that at embryonic day 14 in mice NSCs are committed for a neuronal fate) results into premature appearance of GFAP^+^-glial cells at embryonic day 16 (Stipursky et al., 2014). Thus, we conclude that astrocyte development in early human neural stem cultures (30–40 days post-iPSC stage) does not occur, however, abnormal early glial development can be induced by TGF-β1. Furthermore, we surmise that other factors are required for glial maturation, however, glial maturation factors might be absent in early human neural stem cultures, and thus, the TGF-β1-induced GFAP-glial cells have a rather immature morphology.

### TGF-β Signaling in Electrophysiological Maturation of Neurons and Circuit Formation

Since TGF-β signaling has been reported to influence the balance of excitatory/inhibitory transmission and synaptic plasticity (Sun et al., 2010; Caraci et al., 2015), and given by the here presented TGF-β1 mediated increase of neurons and glial cells, we assessed if TGF-β1 treatment has functional consequences on individual neuron and neuronal network electrophysiological properties.

Presented patch-clamp data revealed no influence on the balance of excitatory/inhibitory transmission. However, we describe (i) a tendency of increased number of neurons with spontaneous action currents and synaptic activity, presented MEA data showed (ii) a tendency of increased number of spiking neurons and (iii) neuronal populations showed more partial synchronous neuronal network activity in TGF-β1-treated cultures in comparison to untreated control cultures. It is thus possible that TGF-β1 increases the number of electrophysiologically active neurons, and thereby, the resulting neuronal population is more active and is more prone to generate population bursts. However, since we did not observe differences in parameters for neuronal excitability and synaptic activity and we did not observe formation of highly synchronously active neuronal networks in TGF-β1-treated cultures and untreated control cultures, we conclude that TGF-β1 by itself does not promote neuronal maturation or processes, such as synapse development, required for neuronal network formation. As described in Figure 1, TGF-β1 is specifically up-regulated during the early phases of human fetal brain development and its expression profile is reciprocal to the synaptic post-synaptic density protein (PSD-93 aka DLG2 (disc large homolog2) and to the neurotrophic factor, e.g., BDNF. Thus, to achieve synchronously active human neuronal networks in TGF-β-treated human iPSC-derived 3D NA cultures requires the application of factors, e.g., BDNF and GDNF, which promote processes involved in neuronal maturation, such as synaptogenesis (Krieglstein et al., 1998; Izsak et al., 2019).

We showed that commonly used neurotrophic factors (BDNF, GDNF, NT-3, and FGF18) support neuronal maturation in human ESC and iPSC-neural cultures (Koch et al., 2009; Kim et al., 2011; Kirkeby et al., 2012; Bardy et al., 2015) and enhance the number of neurons. However, here we reveal that these factors do not suppress neural proliferation. Indeed, neurotrophic factors promote proliferation of neural stem/progenitor cells in rodent-based *in vivo* and *in vitro* models (Bartkowska et al., 2007; Islam et al., 2009). In previous studies, we showed that hiPSC 3D-NAs form highly synchronously active neuronal networks *in vitro* when exposed to neurotrophic factors, e.g., BDNF and GDNF (Izsak et al., 2019). Thus, we conclude that application of neurotrophic factors is not beneficial to prevent proliferation. However, they are rather required for processes involved in neuronal maturation and neuronal circuit formation, e.g., synapse development, and thus, they are predominantly expressed at later stages of human fetal brain development (see Figure 1) and are required to achieve highly functional human iPSC-neuronal networks *in vitro* (Izsak et al., 2019).

### Limitations and Outlook

In the presented study, we focused on analyzing the impact of TGF-β1, and not TGF-β2 or β3. Since different NSC populations *in vivo* and *in vitro* show different response to e.g., TGF-β1 and β2, it will be interesting to evaluate if and how TGF-β2 and β3 are influencing human NSC/progenitor proliferation and differentiation. Interestingly, TGF-β1, but not TGF-β2, suppresses rodent NSC proliferation (Wachs et al., 2006).

We have not experimentally assessed if cell death, e.g., apoptosis, is involved in the reduced number of total cells after TGF-β1 application (Unsicker and Krieglstein, 2002). Given the increase of neuronal and glial cells after TGF-β1 application, we believe that the reduction of total number of cells is rather due to differentiation processes than due to TGF-β1-mediated cell death. Nevertheless, if and when TGF-β mediated cell death occur during human NSC development *in vitro* represents an interesting question for future studies.

The presented QPCR data provide valuable complementary data sets confirming expression of TGF-β ligands and receptors in human iPSC-NSC and 3D-NA cultures. However, comparing the expression profile of TGF-β ligands and receptors obtained from those *in vitro* models with the expression profile of TGF-β ligands and receptors observed during human fetal development reveal major differences. This is not surprising because ours and others human iPSC-NSC and 3D neural *in vitro* models do not show terminal adult-like maturation and truly regional neural identity is absent (see e.g., Bhaduri et al., 2020).

Even though endogenous differentiation factors, e.g., TGF-β1, are present in human iPSC neural cultures, which explain spontaneous neuronal differentiation *in vitro*, we provide evidences that those factors are not sufficiently present in human iPSC-neural models to achieve terminal differentiation and maturation. Thus, supplementing cultivation media with TGF-β1, or other factors, represents a reasonable approach to mimic a physiological environment comprised of neuronal and glial differentiation factors derived from extracerebral sources to improve the physiological relevance of *in vitro* human neural developmental models.

Recently, CSF-like fluid producing human iPSC-derived organoids have been introduced (Pellegrini et al., 2020). Pellegrini et al. showed a dynamic secretome profile over time, and detected a higher abundance of TGF-β1 in early phase organoids, compared to late phase organoids, with a similar trend in the analyzed *in vivo* developing and adult CSF samples (see Figure 5G in Pellegrini et al., 2020). The combination of *in vitro* secreted CSF-like fluid with the more complex cortical organoids represent a promising approach to study human brain development *in vitro* as well as promote the differentiation and functional maturation of human brain organoid models.

Most likely TGF-β1 is not the only signaling cue involved in the control of NSC proliferation, induction of neuronal and glial differentiation during human fetal brain development. Nevertheless, we demonstrated that *in silico* analysis of existing databases and literature followed by the evaluation of candidates in a human neural *in vitro* model system represents an efficient approach to identify signaling cues with physiological relevance for human neural development (Supplementary Figure 3). By presenting our approach, we attend to encourage the search of other natural signaling proteins to enhance the physiological relevance of signaling environment in human neural *in vitro* models.

## Experimental Procedures

### Ethics Statement

We confirm that the experimental procedures were carried out in accordance with regulations and were approved by the named institutions. Work with human iPSC lines was approved by the local ethics committee (Regionala etikprövningsnämnden i Göteborg, DNR 172-08).

### Assessment of Gene Expression Patterns in Human Embryonic and Fetal Brain Development

The human brain transcriptome database was used to assess the gene expression profiles in the *in vivo* human brain development, available at http://hbatlas.org/ (Kang et al., 2011). Only data for neocortical areas were used (“Gene search,” Brain structure = “neocortical areas”). The detailed procedures for data collection have been published (Kang et al., 2011). A detailed description about the process starting from tissue sampling till plotting the diagrams are described here: https://hbatlas.org/files/nature10523-s1.pdf. According to the database, the exon-level transcriptome data was generated using the Affymetrix GeneChip Human Exon 1.0 ST Arrays. The signal intensity for all probes were averaged to obtain an expression value for the probe set. The median of all probe sets within one gene (transcript cluster) was used as the estimate of gene expression. The probe set signal intensity represents the exon expression level (Kang et al., 2011).

### Generation of iPSC Lines, Neural Induction and 3D Neural Aggregate Cultures

Abdominal subcutaneous adipose tissue was isolated and primary adipocyte cell lines were established, as described earlier (Hayashi et al., 2015). HiPSC lines C1 and C2 were generated and characterized by Cellectis (formerly Cellartis, now Takara Clontech). The Cellartis DEF-hiPSC^TM^ ChiPSC4 line was used as an additional commercial line. All lines were cultured under feeder-free conditions in Cellartis DEF-CS^TM^ (Takara Bio Europe AB) at 37°C in a humidified atmosphere of 5% CO_2_ in air. Neural induction was performed by applying the DUAL-SMAD inhibition protocol (Shi et al., 2012), and the detailed neural differentiation procedure for iPSC lines is described in our previous studies (Vizlin-Hodzic et al., 2017; Izsak et al., 2019). Cryostock of hiPSC-NSC cultures were generated by passaging cells by using Accutase 24–30 days after neural induction in hiPSC cultures. Cell suspensions of hiPSC-NSC were stored in 10%-DMSO solution (dissolved in DMEM/F12) and cryostocks were kept at -152°C. Frozen stocks of hiPSC-NSC were thawed and 1.0 × 10^6^ cells were cultured in neural culture media without retinoic acid on Poly-L-Ornithine (PLO) (0.01 mg/ml)/mouse laminin (20 μg/ml) or biolaminin 521 (5 μg/ml) coated 3.5 cm culture plates. Neural culture media comprise of 1:1 mixture of N2 media (DMEM/F12 GlutaMAX, N2 supplement, 5 μg ml^–^1 insulin, 1 mM Ultra glutamine, 100 μM non-essential amino acids, 100 μM 2-mercaptoethanol, 50 U ml^–1^ penicillin and streptomycin (Pen/Strep) and B27 media, Neurobasal, B27 with vitamin A, and 2 mM Ultra glutamine, 50 U ml^–1^ Pen/Strep. After 7–10 days, hIPSC-NSC cultures were comprised of neural rosettes and numerous 3D-neural aggregates. 3D-neural aggregates with diameters ≤ 100 μm were transferred manually and seeded on biolaminin 521-coated coverslips or MEAs. For neuronal differentiation, BrainPhys-media supplemented with N2 supplement, B27 with vitamin A, 2 mM Ultra glutamine, 50 U ml^–1^ Pen/Strep, and 200 μM ascorbic acid were used. Half media exchanges were performed twice a week. Optional human BDNF, GDNF, NT-3, FGF8, TGF-β1 (20 ng/ml), DAPT (10 μM) and SB (0.1, 1, and 10 μM) were added.

### Immunocytochemistry and Confocal Imaging

The procedure for immunocytochemistry is described in our previous study (Izsak et al., 2019). Confocal imaging was performed by LSM 510 META or LSM 710 META (Zeiss). 5 μm optical slices were collected with confocal laser scanning microscopes to visualize NSC (Nestin, CD133, PAX-6), neurons (MAP-2AB), glial cells (GFAP) and proliferating cells (Ki-67) as well as TGF-β receptors I and II. The used primary and secondary antibodies are summarized in Supplementary Table 1. For the evaluation of MAP2AB^+^ neurons in NSCs, either one image per coverslip was taken with the 20x objective, or a tile scan image (4 tiles) with the 40x objective, depending on the cell density. For the evaluation of neural aggregates, 5 individual aggregates were imaged per coverslip using the 40x objective, with 2 individual z levels (10 and 30 μm) to capture the bottom and middle level. The number of MAP2AB^+^ neurons were manually quantified using Cell Counter in ImageJ and normalized per number of total DAPI nuclei. The DAPI^+^ cell nuclei and Ki-67^+^ nuclei, were quantified by the Nucleus counter plugin (Schneider et al., 2012). For the quantification of GFAP^+^ area coverage, one image per coverslip was taken with an 10x objective, and the percentage of area covered across the image was measured in ImageJ then further normalized per DAPI nuclei. All the image quantification was manually revised to exclude false signal detection. All experiments have been repeated two to three times with duplicates or triplicates for each marker per experiment (see figure legends for details).

### RNA Extraction and cDNA Synthesis

Total RNA was extracted and contaminating genomic DNA was eliminated using the RNAeasy^®^Micro Kit (Qiagen) according to manufacturer’s instructions. Total RNA concentrations were measured using Qubit RNA HS Assay Kit on a Qubit 4 Fluorometer (Thermo Fisher Scientific). cDNA was synthesized from 500 ng of total RNA using a RevertAid H minus First Strand cDNA synthesis kit (Thermo Fisher Scientific) in a total reaction volume of 20 μl.

### Quantitative PCR

Quantitative PCR was performed using TaqMan Gene Expression Assays with FAM reporter dye (TGFB1: Hs00998133_m1, TGFB2: Hs00234244_m1, TGFB3: Hs01086000_m1, TGFBR1: Hs00610320_m1, TGFBR2: Hs00234253_m1, TGFBR3: Hs00234257_m1, GFAP: Hs00909233_m1) in TaqMan Universal Master Mix II with UNG in a total reaction volume of 25 μl on a Step One Plus Real Time PCR System (Applied Biosystems). The relative quantity of gene expression was determined using the ΔCT method, with HPRT1 (Hs02800695_m1) as endogenous reference. HPRT1 (Hs02800695_m1) was used as endogenous reference since its expression was neither affected by maturation nor TGF-β1 treatment.

### Cell-Attached and Whole-Cell Recordings

For electrophysiological experiments, frozen stocks of hIPSC-NSC were cultured on PLO/laminin-coated plates to enrich cell number. After 7 days, cells were passaged and plated on PLO/laminin-coated Ibidi μ-dishes (Ibidi) and maintained in BrainPhys culture media comprising supplements, as described before, for up to 22 days in the presence or absence of TGF-β1 (20 ng/ml). The μ-dishes were mounted under a microscope (Nikon E600FN), where the cells were perfused (2–3 ml/min) with artificial CSF (ASCF) containing: 1 mM NaH_2_PO_4_, 123 mM NaCl, 26 mM NaHCO_3_, 3 mM KCl, 2 mM MgCl_2_, 1 mM CaCl_2_, and 10 mM D-glucose. The ACSF was continuously bubbled with gas containing 95% O_2_ and 5% CO_2_. Patch-clamp recordings were performed on cells at the edge of 3D-neural aggregates and visually identified using infrared differential interference contrast video microscopy. Recordings and data analysis are identical to our previous studies (Izsak et al., 2019, 2020).

### Multi-Electrode Array Recordings and Pharmacological Experiments

Frozen stocks of hIPSC-NSC were cultured as a 5 μl drop directly on biolaminin 521 coated electrode array of 6-well multi-electrode arrays (MEAs). After 1 h, 200 μl BrainPhys^TM^ media with supplements (described above) was added. Optional TGF-β1 (20 ng/ml) was used. Half media exchanges were performed twice per week. MEAs had a square grid of 9 planar Ti/TiAu electrodes with PEDOT-CNT (carbon nanotube poly-3,4-ethylene-dioxythiophene) of 30 μm diameter and 200 μm spacing. Recordings have been performed in BrainPhys media supplemented with B27, N2, and L-glutamine. Details about the used MEA set-up and data analysis are described in our previous studies (Izsak et al., 2019, 2020).

### Statistical Analysis

For statistical analysis either one-way ANOVA with Tukey’s correction (multiple comparisons) or unpaired *t*-tests implemented in GraphPad prism (version 8) were used. All presented data show mean value ± SD. N refers to the number of individual experiments, n refers to the number of technical replicates.

## Data Availability Statement

The data that support the findings of this study are available from SI, upon reasonable request send to sebastian.illes@neuro.gu.se.

## Author Contributions

JI performed most of the experiments, data analysis, prepared all figures, wrote part of the manuscript. MI and JJ performed the QPCR experiments and analysis. DV-H performed neural differentiation of ChiPS4 hiPSC-lines. JS and TOB performed patch clamp experiments and data analysis. ST developed data analysis programs and critically revised the manuscript. EH, HÅ, and KF critically revised the manuscript. SI conceived the study, performed part of the experiments, and wrote the manuscript. All authors contributed to the article and approved the submitted version.

## Conflict of Interest

SI holds a position at the company Cellectricon. Cellectricon was not involved in the study, and all experiments and data analysis were conducted at the Sahlgrenska Academy at the University of Gothenburg. ST was founder of the company Result Medical GmbH, Düsseldorf, Germany. JJ holds a position at the company Kyowa Kirin GmbH. Kyowa Kirin GmbH was not involved in the study, and all experiments and data analysis were conducted at the University of Düsseldorf. The remaining authors declare that the research was conducted in the absence of any commercial or financial relationships that could be construed as a potential conflict of interest.
