# Supplementary figures and images for: TGF-β1 Suppresses Proliferation and Induces Differentiation in Human iPSC Neural in vitro Models
Source: Front Cell Dev Biol. 2020 Oct 28;8:571332. doi: 10.3389/fcell.2020.571332 (PMC7655796; doi:10.3389/fcell.2020.571332)

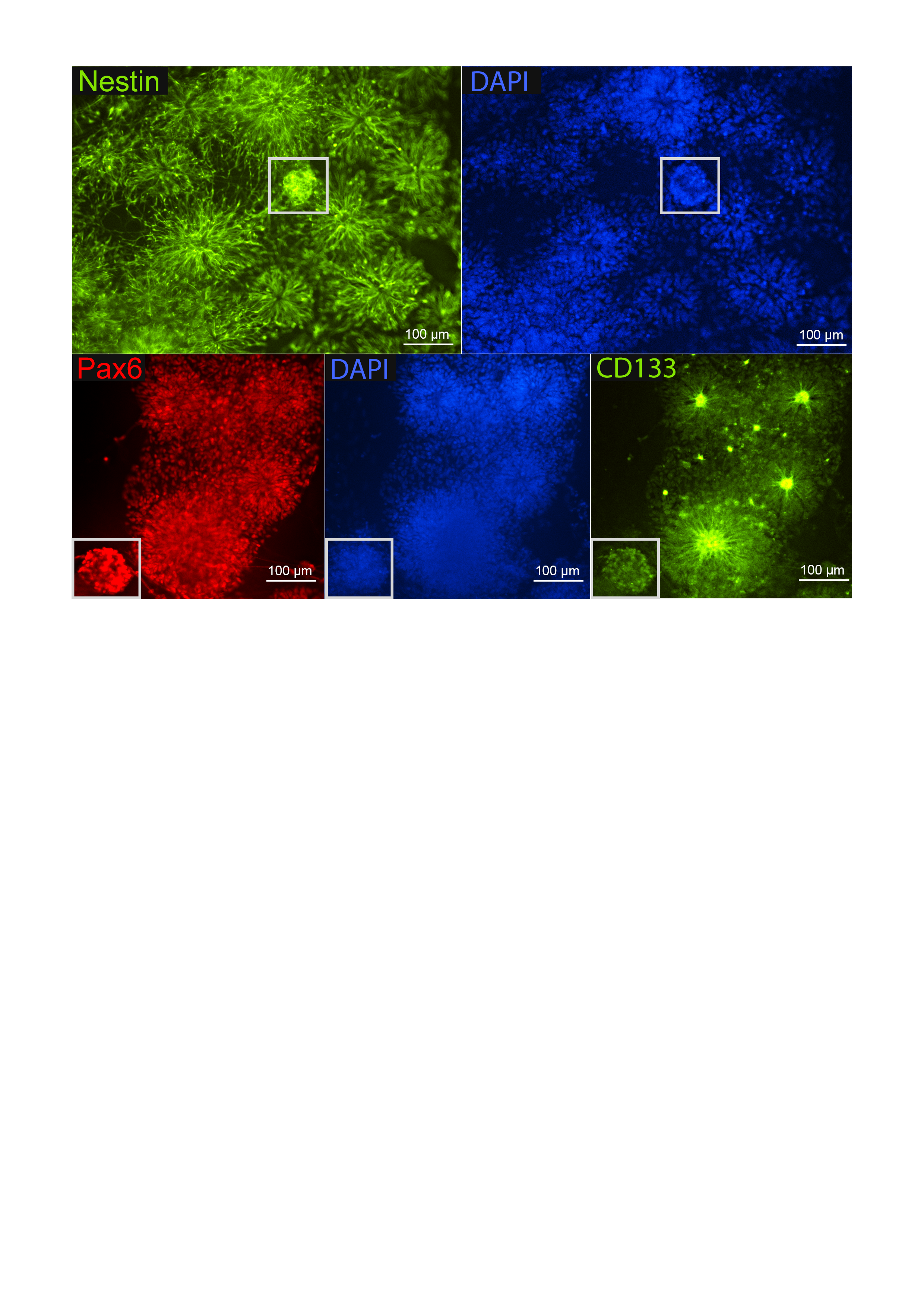

Supplement: Supplementary file 1 [file Image_1.TIF]

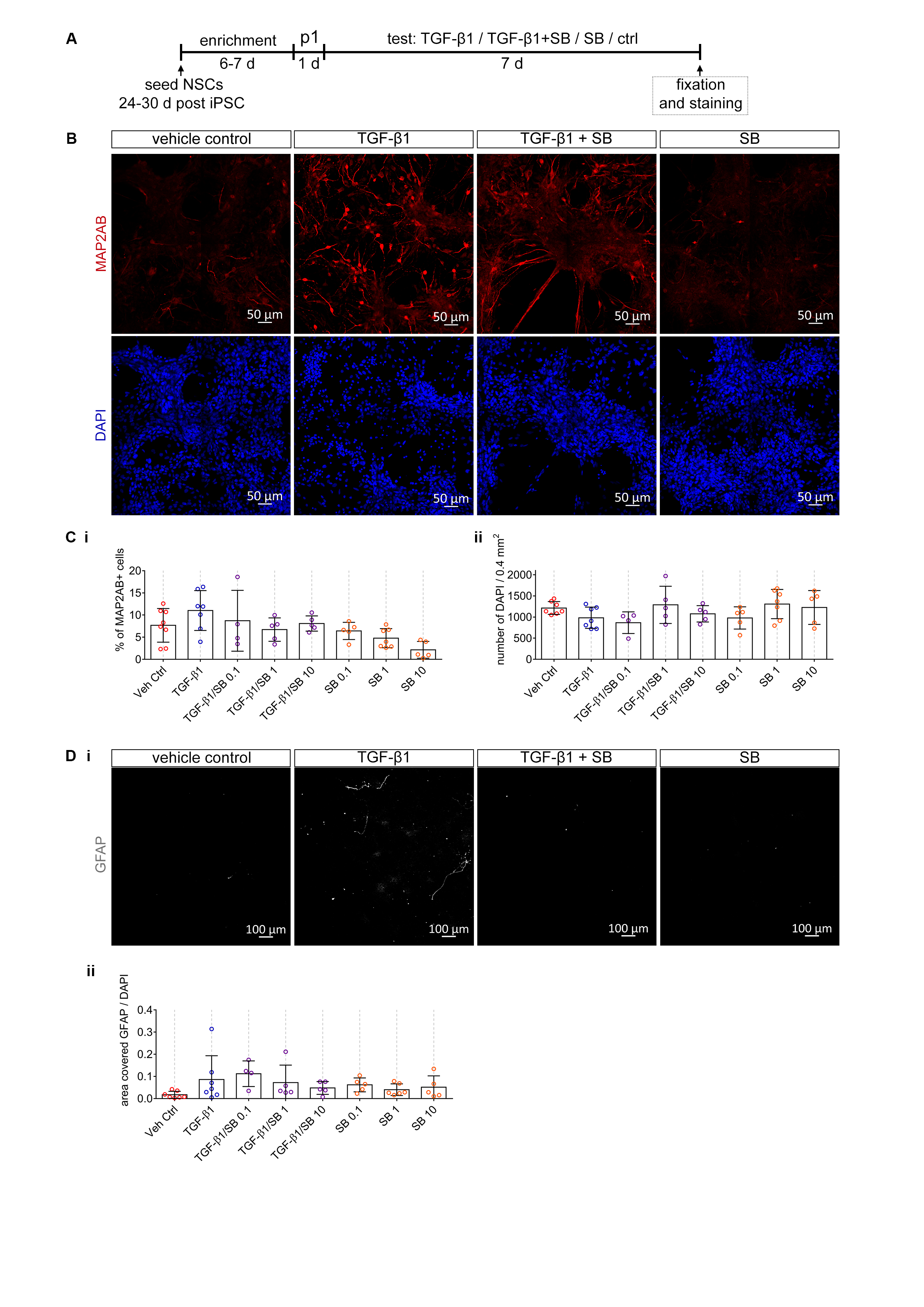

Supplement: Supplementary file 2 [file Image_2.TIF]

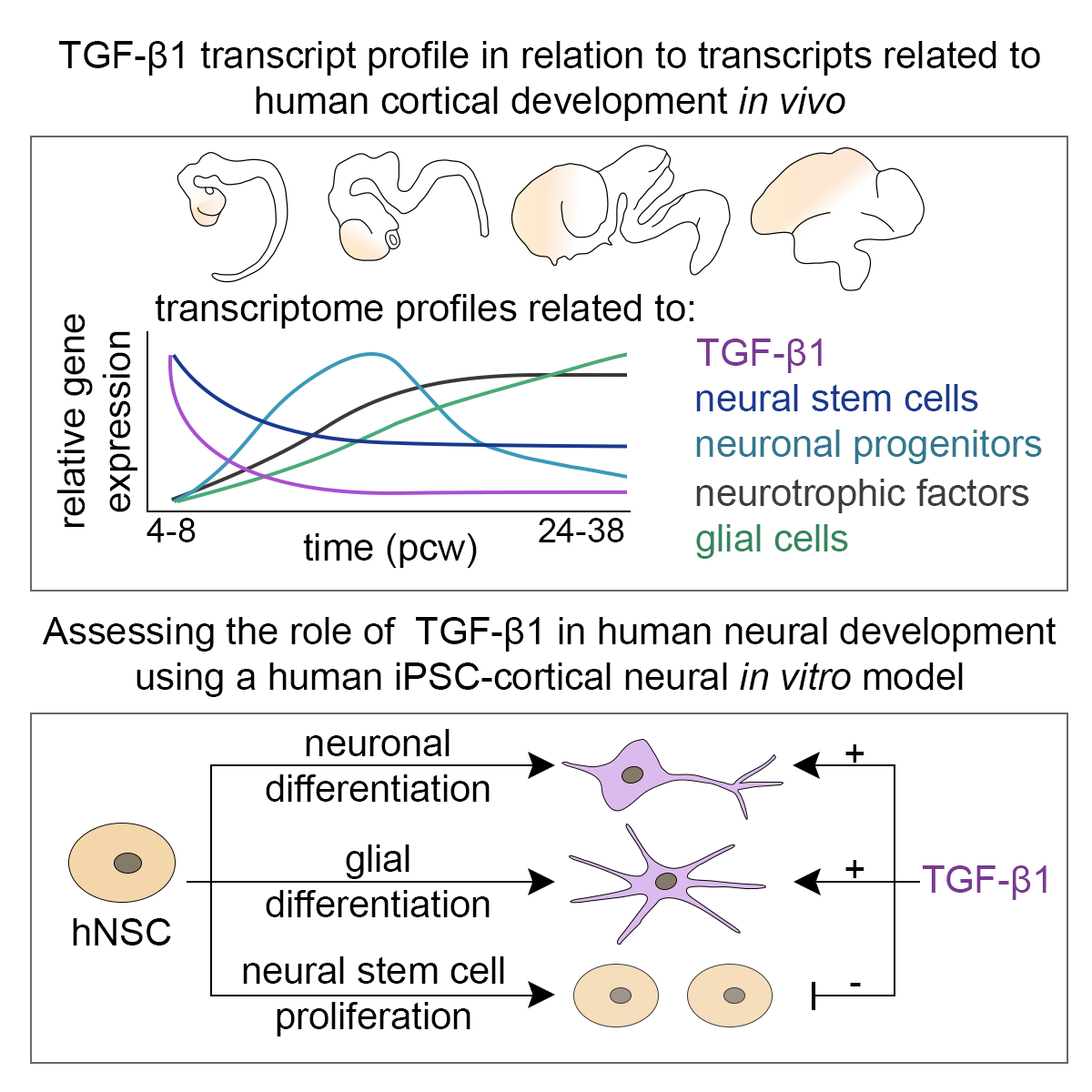

Supplement: Supplementary file 3 [file Image_3.TIF]
