# Supplementary material for: TGF-β1 Suppresses Proliferation and Induces Differentiation in Human iPSC Neural in vitro Models
Source: Front Cell Dev Biol. 2020 Oct 28;8:571332. doi: 10.3389/fcell.2020.571332 (PMC7655796; doi:10.3389/fcell.2020.571332)
Supplement: Supplementary file 4 [file Table_1.DOCX]

| **Antibody** | **Supplier (cat. No.)** | | **Host** | | **Dilution** |
| --- | --- | --- | --- | --- | --- |
| Nestin | Merck (MAB353) | | mouse | | 1:500 |
| CD133 | Miltenyi Biotec (130-090-422) | | mouse | | 1:500 |
| Pax6 | Millipore (ab2237) | | rabbit | | 1:200 |
| MAP2AB  MAP2AB | Abcam (ab11267)  Abcam (ab5392) | | mouse  chicken | | 1:1000  1:2000 |
| Ki-67  GFAP | Santa Cruz (sc-15402)  Abcam (ab7260) | | rabbit  rabbit | | 1:500  1:500 |
| TGF-β RI | Santa Cruz (sc-398) | | rabbit | | 1:250 |
| TGF-β RII | Santa Cruz (sc-400) | | rabbit | | 1:250 |
| Alexa Fluor 488 | Thermo Fisher (A11029) | | goat anti-mouse | | 1:500 |
| Alexa Fluor 555  Alexa Fluor 555 | Thermo Fisher (A31572)  Thermo Fisher (A32932) | | donkey anti-rabbit  goat anti-chicken | | 1:500  1:500 |
|  |  |  | |  | |
